# Does suboptimal household flooring increase the risk of diarrhoea and intestinal parasite infection in low and middle income endemic settings? A systematic review and meta-analysis protocol

**DOI:** 10.1186/s13643-020-01384-9

**Published:** 2020-05-20

**Authors:** Benn Sartorius, Hugo Legge, Rachel Pullan

**Affiliations:** 1 Department of Disease Control, Faculty of Infectious and Tropical Diseases, London School of Hygiene and Tropical Medicine, London, WC1E 7HT UK; 2 Department of Health Metrics Sciences, School of Medicine, University of Washington, Seattle, WA USA; 3 Department of Public Health Medicine, University of KwaZulu-Natal, Durban, South Africa

**Keywords:** Diarrhoea, Intestinal parasite, Infections, Risk factors, Unimproved household flooring, Low- and middle-income country

## Abstract

**Background:**

Water, sanitation, and hygiene interventions often fail to show long-term impact on diarrhoeal and/or intestinal parasite risk in many low- and middle-income countries. Less attention has been paid to wider contextual factors that may contribute to high levels of contamination in the domestic environment such as household flooring. The purpose of this study will be to assess the association between diarrhoeal and/or intestinal parasite infection status and unimproved/unfinished flooring in low- and middle-income countries.

**Methods:**

We will conduct a comprehensive search of published studies (randomized controlled trials, non-randomized controlled trials, and observational studies) that examined the association between unimproved/unfinished household flooring and diarrhoeal and/or intestinal parasite infection status from January 1, 1980, onwards with no language restriction. The primary outcome will include diarrhoeal and/or intestinal parasite infection status. Databases to be searched include EMBASE, MEDLINE, Web of Science, and Google Scholar. The secondary outcome will be the association between specific pathogens (laboratory confirmed) and unimproved/unfinished household flooring. Independent screening for eligible studies using defined criteria and data extraction will be completed in duplicate and independently. Any discrepancies between the two reviewers will be resolved by consensus and/or arbitration by a third researcher. If data permits, random effects models will be used where appropriate. Subgroup and additional analyses will be conducted to explore the potential sources of heterogeneity (e.g. age group, geographical region) and potential risk of bias of included studies.

**Discussion:**

This review will provide a comprehensive examination of a possible association between suboptimal household flooring and increased risk of enteric pathogen infection, highlight gaps for future research in high risk areas, and inform intervention design for future planned studies in Kenya and/or elsewhere in the region.

**Systematic review registration:**

PROSPERO registration number: CRD42019156437

## Background

Enteric parasites and diarrhoeal disease are amongst the most widespread diseases in low- and middle-income settings [1–3], with an attributed 330,000 deaths due to diarrhoea alone amongst children in sub-Saharan Africa (SSA) in 2015 [4]. A significant proportion of vulnerable children in SSA remains at risk for contracting enteric disease due to the presence of multiple risk factors facilitating their transmission such as inadequate sanitation, unsafe water sources, poor hygiene practices, and poverty [5].

The relationship between water, sanitation, and hygiene (WASH) and enteric/parasitic disease is complex. Global estimates suggest that improvements in water and sanitation have contributed to reductions in enteric disease burden [6]. However, WASH interventions frequently fail to show long-term impact [7], due to re-infection, poor uptake, and use. Other suggested explanations include contamination throughout the home as a result of dirt floors [8, 9]. Contaminated household flooring can facilitate increased contamination of hands, food, and objects that encounter these surfaces and potentially increase risk of diarrhoea and parasitic disease [10]. Less attention has been paid to wider contextual factors that may contribute to the burden of enteric pathogens, particularly those that contribute to high levels of contamination in the domestic environment such as household flooring. Sand, soil, or other rudimentary household floors are hard to clean and sanitise and can remain damp, providing breeding grounds for pathogens. This can be further exacerbated by rainfall, season, local terrain, soil type, soil moisture, water saturation, and relative atmospheric humidity [11–14] and is especially problematic when environmental contamination is high, for example in communities where animals live in close proximity to humans [15]. Unhygienic floors may thus have important health impacts discrete from other established risk factors.

There is increasing interest in ‘healthy homes’ and infectious disease, which at this stage has been primarily focused on vector control; however, there is an urgent need to assess the relationship between the built environment and its impact on enteric infection risk. While some observational studies have suggested no association between unimproved flooring and enteric pathogen risk, multiple observational studies have shown associations between unimproved flooring and diarrhoeal risk [16] as well as some enteric parasitic infections, notably STH [9]. However, no systematic review of the literature has been conducted, to our knowledge, to assess whether unimproved household flooring is an independent risk factor for enteric disease risk in low- and middle-income countries (LMIC). The suggested association between unimproved household flooring and increased risk of enteric/parasitic infection may simply be due to confounding by SES; however, this relationship has not been sufficiently explored.

To foster the design of more effective integrated control strategies, the main aim of this systematic review and meta-analysis will be to assess the association between enteric pathogen infection and sub-optimal or unimproved household flooring (adjusted for other key risk factors) through pooled risk estimates using all available evidence from LMIC.

## Methods

### Patient and public involvement statement

Patients were not involved in the development of this protocol.

### Study design

The present protocol has been registered within the PROSPERO database (registration number CRD42019156437) and is being reported in accordance with the reporting guidance provided in the Preferred Reporting Items for Systematic Reviews and Meta-Analyses Protocols (PRISMA-P) statement [17], which informed the development of this protocol (see checklist in Supplementary Material 1).

### Information sources

1. The following minimal set of electronic databases of peer-reviewed literature as suggested by Bramer et al. [18] will be systematically searched with no language restriction and restricted to studies of humans only: EMBASE, MEDLINE, Web of Science, and Google Scholar. Electronic searches will be additional complimented by a manual search of reference lists of identified articles.
2. Study authors will be contacted where relevant for clarification or for additional missing key meta-data.
3. Google Scholar will be used to search for grey literature including relevant unpublished data and relevant websites such as randomized controlled trial registries, Global NTD database, and conference proceedings quantifying the association between unimproved/unfinished household flooring and diarrhoea/intestinal parasitic infection status.
4. Studies conducted from January 01, 1980-onwards, with no language restriction, will be eligible for inclusion. Furthermore, only studies conducted in a low- and middle-income setting will be eligible for further screening.

### Search strategy

When searching PubMed/Medline, Medical Subject Headings (MESH) search terms will be used. Search terms to be used will include keywords referring to ‘household flooring’, ‘floor’, ‘dirt’, ‘earthen’, ‘cement’, ‘wood’, ‘tile’, ‘concrete’, ‘hard’, ‘solid’, ‘enteric’, ‘diarrhea’, ‘diarrhoea’, ‘soil-transmitted’, ‘helminth’, ‘worm’, ‘intestinal parasite’, ‘giardia’, ‘entamoeba’.

The following proposed search term will be used: “(soil OR dirt OR earth* OR cement OR wood OR tile OR concrete OR hard OR solid) AND floor* AND (soil-transmitted OR “soil transmitted” OR enteric OR diarrh* OR intestinal OR protozoa) AND (infection OR helminth* OR worm OR parasit*)”. A draft search for PubMed/MEDLINE is available in Supplementary Material 2.

### Eligibility criteria

Studies will be selected or excluded for inclusion based on the criteria proposed in Table 1. Study published from 1 January 1980 onwards will be eligible for inclusion. We will not apply any language restrictions as part of the eligibility criteria.

**Table 1:** Inclusion and exclusion criteria

Category	Included	Excluded
**Population**	Children and adults residing in low- and middle-income country (based upon World Bank country classification).	Children and adults residing in a high-income country (based upon World Bank country classification); livestock or pets
Intervention/**exposure and comparator**	Household flooring: unfinished or natural (earth, soil, sand, clay, mud, dung) versus finished (polished wood, tiles, cement, stone, bricks) Confounders: unimproved drinking water (e.g. unprotected spring, well, or surface water) and/or sanitation (e.g. shared latrine, pit latrine without slab, hanging toilet or hanging latrine, flush/pour flush to elsewhere, bucket, no facilities, or bush or field), low socio-economic status	Studies not assessing household flooring type as an exposure
**Study design**	Quantitative studies including: randomized and non-randomized controlled trials, cohort, cross sectional, case-control	Qualitative studies (e.g. on process and perception of interventions); quantitative studies not measuring exposure or outcome status
**Outcome**	Diarrhoea and/or presence of intestinal parasites (soil-transmitted helminth or protozoa) Secondary outcomes: diarrhoeal pathogen species (if laboratory confirmed) and/or intestinal parasite species (if laboratory confirmed)	Non-enteric infection or parasitism

The PICO (Population, Intervention, Comparison, Outcome) classification approach is widely used as a strategy for formulating search strategies and characterizing meta-analyses. We have utilised this framework to explicitly define the criteria for eligibility (Table 1). Studies including children and adults residing in low- and middle-income country (based upon World Bank country classification) will be eligible for inclusion. The primary outcome will be self-reported diarrhoea (usually defined as three or more loose or watery stools in 24 h, as reported by the mother/caretaker of the child in the preceding 2 weeks), laboratory confirmed presence of a diarrheal pathogen (stool examination or culture) where available, and/or laboratory confirmed presence of intestinal parasites (microscopy or serology or molecular). The primary exposure will be residing in a household with unfinished flooring (e.g. dirt, soil, or earth) compared with finished flooring (e.g. cement or concrete, tiled). If available, we will also extract multivariable adjusted measures of association between unimproved/improved flooring and diarrhoea/intestinal parasitic infection status adjusted for household water/sanitation quality and socio-economic standing. Unadjusted measures of association will be extracted but will be assigned a lower quality weighting (please see ‘Risk of bias assessment for eligible studies’ section). We will include randomized and non-randomized controlled trial studies with baseline assessment of the exposure-outcome of interest and analytic observation studies (namely cohort, cross sectional, case-control).

### Screening and selection procedure

Retrieved citations from the various search engines will be imported into EndNote and checked for duplicates, i.e. de-duplicated. Eligibility of the studies will be ascertained by two independent researchers who will screen the titles and abstracts (phase 1). Full articles of potentially eligible titles/abstracts will be independently assessed by two researchers (BS and HL) for inclusion in the review (phase 2). In case of disagreement about the eligibility, a third investigator (RP) will be consulted. Articles will be archived in a data extraction spreadsheet that will be pilot-tested to evaluate its appropriateness.

### Data extraction

The data capture sheet will consist of a screening checklist consisting of study details (author, year of study, year of publication, type of publication, country in which study was carried out), study characteristics (study design, sample size, mean age, and age range of participants), measures of exposure (household flooring, sanitation facilities, drinking water source, socio-economic status) and outcome/disease status (diarrhoea and/or presence of intestinal parasites status as well as specific species if laboratory confirmed) (Table 1), and measures of the association between unfinished/unimproved household flooring and diarrhoea and/or presence of intestinal parasites status (odds ratios, prevalence ratios, relative risks or hazard ratios), with their related variability (standard deviations or errors [SD, SE respectively), and 95% confidence intervals [95% CI]). Please see ‘Eligibility criteria’ section and Table 1 for the list and definitions of the aforementioned exposures and outcomes. Furthermore, we will also extract whether the measure of association was adjusted for household sanitation facility type, drinking water source, and/or socio-economic status and which of the aforementioned confounders where adjusted for explicitly. Any notable study limitations will be extracted and other additional relevant information requested from the corresponding author(s) as required.

### Data synthesis and additional analyses

#### Outcome and prioritization

The primary outcome to be analysed will be any diarrheal/intestinal parasite infection. As a secondary outcome, we will also attempt to assess the association between specific pathogens and unimproved/unfinished household flooring for the subset of studies for which this was laboratory confirmed (if numbers of eligible studies are sufficient).

To accurately report on the content of individual eligible papers and to explore relationships between the outcome(s) and risk factor(s), data will be summarised in tabular format presenting the main findings of each paper individually, including population under study, region or continent (Sub-Saharan Africa or Africa, South America, Central America, Asia), outcomes (diarrhoeal and/or intestinal parasite infection status), exposures (specific household flooring type), and main results (number of cases, rates or prevalence, odds ratios or relative risks). A qualitative or narrative summary of the key characteristics and quality of the papers will also be included, and in addition, we will also present the risk of bias summary for each paper (please see ‘Risk of bias assessment for eligible studies’ section).

As epidemiological and clinical heterogeneity is expected, all meta-analyses in this study will be conducted using the random effects model. We will employ random effects modelling as the effect(s) underlying the different studies are assumed to be drawn from a normal distribution, considering both within- and between-study variation. Forest plots will be used to visually assess pooled estimates and ascertain the extent of heterogeneity between studies. We will quantify statistical heterogeneity by estimating the variance between studies using I^2^ statistic [19]. The I^2^ statistic is the proportion of variation in prevalence estimates that is due to genuine variation in treatment effects rather than sampling (random) error. I^2^ statistic ranges between 0 and 100% (with values of 0–25% and 75–100% taken to indicate low and considerable heterogeneity, respectively) [20]. We will also report Tau^2^ [21] and Cochran’s Q test [22] with a *P* value of < 0.05 considered statistically significant (heterogeneity).

If the selected studies are deemed suitable for quantitative synthesis, data will be pooled in a meta-analysis approach combining the primary exposure (unimproved household flooring) across studies and presented as a summary of effects based on study design/measure of effect, e.g. odds ratios versus relative risk.

If a meta-analysis approach is justified, we will likely employ random effects models with inverse variance weighing to estimate the pooled/meta-weighted effect size for unimproved/unfinished household flooring versus diarrheal/intestinal parasite status across eligible studies with 95% CI. If there are sufficient studies/data, we may also conduct a meta-regression analysis utilising various other meta-covariates extracted from the eligible. The analysis will be conducted using the R statistical software (R core team (2019). R: A language and environment for statistical computing. R Foundation for Statistical Computing, Vienna, Austria. URL http://www.R-project.org/) or STATA/IC V 16 (StataCorp. 2019. Stata Statistical Software: Release 16. College Station, TX: StataCorp LLC).

Where possible, we will also perform subgroup analysis by age group, study design (i.e. cross-sectional or prevalence studies, case-control, cohort), and/or geographical region (Africa, Central-South America, Asia), in order to assess differences between the strength of association by geography and potential impact of contextual confounders which may vary by geography. This will also be extended to a subgroup analysis across specific pathogen species if there are a sufficient number of studies.

### Risk of bias assessment for eligible studies

This systematic review can be categorised under aetiology and risk (exposure and outcome). We will use the Cochrane Risk of Bias (RoB) 2.0 Tool for assessing risk of bias for randomised controlled trials [23]. We will use the Newcastle–Ottawa Scale (NOS) to assess the risk of bias for observational studies [24]. Each study will be assessed individually and independently by the two reviewers, both at outcome and study level to generate an overall risk of bias score. Each reviewer will assign each study as ‘low’, ‘moderate’, ‘serious’, or ‘at critical’ risk of bias. The quality of evidence for an association between unimproved/unfinished household flooring and diarrhoeal and/or intestinal parasite infection status will be evaluated according to the risk of bias categorisation.

### Meta-biases

Funnel plots [25] will be used to graphically assess if there is potential publication bias and further confirmed using statistical based tests such as Egger’s and/or Begg’s tests [26, 27]. We will also compare the estimate from the fixed effect model against the random effects model to assess the possible presence of small sample bias amongst the published articles (i.e. whether the effect of the exposure is more pronounced in smaller studies).

## Discussion

WASH interventions often fail to show long-term impact on diarrhoeal and/or intestinal parasite risk in many LMIC. Less attention has been paid to wider contextual factors that may contribute to high levels of contamination in the domestic environment such as household flooring. It has been hypothesized that unimproved/unfinished household flooring may be an important contextual risk factor in these settings. This systematic review aims to assess whether there is a significant association between unimproved/unfinished household flooring and the risk of diarrhoea and/or intestinal parasite infection in low- and middle-income settings. We anticipate that differences in floor type classification across different settings and variation in confounder measurement/classification as well as lack of multivariable adjustment may be potential limitations.

The findings of this review will augment current knowledge and add to the evidence base regarding risk factors for diarrhoea and intestinal parasites in low- and middle-income countries (LMIC). If this is found to be the case, it would help identify children at greater risk based on household living conditions that would have important implications for interventional packages and will help inform future interventions and establish whether more research is warranted, and potentially highlight additional intervention measures required to reduce this burden in LMIC.

We do not envisage any amendments to the present protocol. However, should any essential amendments be found to be necessary, they will be reported in the published review.

The results will be disseminated in the form of a peer-reviewed journal article and also be shared with relevant health authorities such as WHO.

## Supplementary information

**Additional file 1: Supplementary Material 1.** PRISMA-P 2015 Checklist.

**Additional file 2: Supplementary material 2.** Draft search using PubMed/MEDLINE, including planned temporal limits.

## Data Availability

Raw meta-data extracted for the purposes of this review will be made publically available with the final published review findings.

## Abbreviations

CI: Confidence interval
LMIC: Low- and middle income country
MESH: Medical Subject Headings
MOOSE: Meta-analysis of Observational Studies in Epidemiology
NTD: Neglected tropical diseases
PRISMA-P: Preferred Reporting Items for Systematic Reviews and Meta-Analyses Protocols
SD: Standard deviation
SE: Standard error
STH: Soil-transmitted helminth
WASH: Water, sanitation, and hygiene
WHO: World Health Organization
