# Supplementary material for: Does suboptimal household flooring increase the risk of diarrhoea and intestinal parasite infection in low and middle income endemic settings? A systematic review and meta-analysis protocol
Source: Syst Rev. 2020 May 20;9:113. doi: 10.1186/s13643-020-01384-9 (PMC7240925; doi:10.1186/s13643-020-01384-9)
Supplement: Supplementary file 2 — Additional file 2: Supplementary material 2. Draft search using PubMed/MEDLINE, including planned temporal limits. [file 13643_2020_1384_MOESM2_ESM.docx]

| Search query: (soil OR dirt OR earth* OR cement OR wood OR tile OR concrete OR hard OR solid) AND floor* AND (soil-transmitted OR "soil transmitted" OR enteric OR diarrh* OR intestinal OR protozoa) AND (infection OR helminth* OR worm OR parasit*) | | |
| --- | --- | --- |
| Year | Count |  |
| 2020 | 5 |  |
| 2019 | 15 |  |
| 2018 | 9 |  |
| 2017 | 10 |  |
| 2016 | 9 |  |
| 2015 | 8 |  |
| 2014 | 7 |  |
| 2013 | 6 |  |
| 2012 | 7 |  |
| 2011 | 9 |  |
| 2010 | 6 |  |
| 2009 | 6 |  |
| 2008 | 4 |  |
| 2007 | 5 |  |
| 2006 | 6 |  |
| 2005 | 4 |  |
| 2004 | 8 |  |
| 2003 | 4 |  |
| 2002 | 4 |  |
| 2000 | 1 |  |
| 1999 | 3 |  |
| 1998 | 7 |  |
| 1997 | 1 |  |
| 1996 | 4 |  |
| 1994 | 3 |  |
| 1992 | 1 |  |
| 1991 | 3 |  |
| 1990 | 1 |  |
| 1989 | 3 |  |
| 1988 | 3 |  |
| 1985 | 1 |  |
| 1984 | 1 |  |
| 1983 | 2 |  |
| 1981 | 2 |  |
| 1980 | 1 |  |
